# Bioprotective and Functional Activities of Postbiotics From Lactic Acid Bacteria Derived From Artisanal Dairy Products

**DOI:** 10.1002/fsn3.70647

**Published:** 2025-07-16

**Authors:** Areeba Tariq, Merve Nur Tahran, Sebnem Ozturkoglu‐Budak

**Affiliations:** ^1^ Graduate School of Natural and Applied Sciences, Ankara University Ankara Turkey; ^2^ Department of Dairy Technology Faculty of Agriculture, Ankara University Ankara Turkey

**Keywords:** bioactivity, bioprotective, cell‐free supernatant, lactic acid bacteria, postbiotics

## Abstract

Postbiotics have been reported as a more stable and safer alternative to live microorganisms for industrial food applications. This study demonstrates the use of cell‐free supernatant and cell‐wall components derived from 10 native Lactic Acid Bacteria (LAB) as a functional and bioprotective agent, emphasizing its potential as a preservative in pasteurized milk. These species were from *Lactiplantibacillus* ( *L. plantarum* , *L. paraplantarum* ), *Loigolactobacillus* ( *L. coryniformis* ), *Levilactobacillus* (*Lb. brevis*), *Lactobacillus* (*Lb. helveticus*, and *Lb. bulgaricus*), *Enterococcus* ( *E. faecium* and *E. faecalis* ), *Lactococcus* (*Lc. lactis*), and *Streptococcus* (*St. thermophilus*) genera. Postbiotics, obtained as cell‐free extract and cell‐wall components, were combined and subjected to biopreservation and in vitro functional analyses, including antioxidant and antimicrobial activities, as well as their phenolic and organic acid contents. To assess their bioprotective effects, these postbiotics were applied to pasteurized milk, both with and without *E. coli* (ATCC 25922). According to the results, *L. plantarum* , *L. paraplantarum* , *E. faecalis* , *E. faecium* , *L. brevis* , and *Lc. lactis* were found to produce high levels of antioxidants and phenolic compounds. This is believed to contribute to the higher antimicrobial activity exhibited by their postbiotics. In pasteurized milk, all postbiotics demonstrated a reduction in the *E. coli* count, highlighting their bioprotective potential. However, this decrease was particularly significant in the presence of postbiotics from *E. faecium* , *Lb. helveticus*, and *Lc. lactis*, which are also determined as producers of acetic and formic acids. It is suggested that the specific desired properties of the final product can guide the selection of the appropriate strain for obtaining postbiotics.

## Graphical abstract

Postbiotics derived from 10 native LAB species were evaluated for their in vitro functional properties and their biopreservative effects in a model product of pasteurized milk. Postbiotics belonging to *E. faecalis* , *Lc. lactis*, *L. plantarum* , and *L. paraplantarum* were accepted as potential candidates in terms of in vitro functional properties. As to biopreservation capacity, *E. faecium* , *Lc. lactis*, and *Lb. brevis* was chosen as the best strain. The preferred microbial source can be selected based on the intended purpose of the product, such as enhancing antimicrobial or antioxidant activity, or increasing phenolic and/or organic acid content.

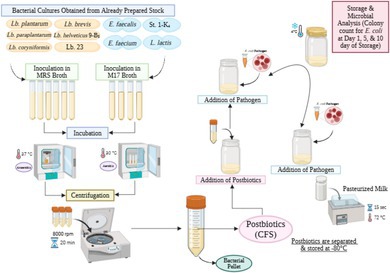

## 1 Introduction

Postbiotics encompass a wide range of metabolites and by‐products with beneficial effects released by different microorganisms during food fermentation processes. These compounds can either be chemical substances produced during normal microbial activity or materials, including cellular fragments of producer microorganisms (Salminen et al. 2021).

The most common method of obtaining postbiotics involves deactivating bacterial cells and suspending the remaining substances in the supernatant. Since the supernatant contains no live bacteria, it is referred to as Cell‐Free Supernatant (CFS). This supernatant is rich in beneficial components such as organic acids, short‐chain fatty acids (SCFAs), peptides, and bacteriocins.

Postbiotics can also be derived by disrupting the producer bacterial cell membrane to obtain its fragments or by extracting intracellular components through cell lysis (O'Toole et al. 2017). Disruption of the bacterial membrane releases various surface‐associated components, including exopolysaccharides, lipoteichoic acids, and glycolipids. In contrast, cell lysis provides both membrane fragments and intracellular substances, which often exhibit properties similar to their source microorganisms. These bioactive components demonstrate a wide range of health benefits, including antimicrobial, anticancer, antiproliferative, antidiabetic, and anti‐inflammatory effects. They also play a crucial role in enhancing the body's immunomodulatory response (Cuevas‐González et al. 2020).

Microbial viability is a fundamental requirement for probiotic microorganisms to exert their biological effects on consumers. For probiotics to be effective, they must be present in the final product at concentrations of approximately 10^6^ to 10^9^ CFU/g or mL throughout production, processing, storage, and even during digestion (Sarkar 2013). However, during industrial production, several factors can reduce the viability of probiotic cells. These include the composition of the food matrix (e.g., pH, protein, fat, and carbohydrate concentration, water activity, and the presence of natural antibiotics) as well as processing and storage conditions, such as time, temperature, inoculation rate, pH, oxygen exposure, and packaging materials (Collado et al. 2019). Studies have also reported that some commercial products contain probiotics at concentrations much lower than those stated on their labels, raising concerns about their efficacy (Fredua‐Agyeman et al. 2016).

The potential side effects of consuming live microorganisms on health have been a topic of discussion. While many studies have reported that probiotics are generally safe, recent research has raised concerns, particularly regarding their use in high‐risk groups such as immunocompromised individuals and cancer patients (Suez et al. 2019). Reported side effects include systemic infections, excessive immune stimulation in susceptible individuals, transfer of virulent genes by probiotic bacteria, gas and bloating due to intestinal overgrowth, and failure to meet safety standards, such as the horizontal gene transfer of antibiotic‐resistant genes within the body (Lerner et al. 2019). Additionally, studies on probiotics have highlighted other concerns, including unknown molecular mechanisms, strain‐specific behaviors, and severe complications such as infective endocarditis, sepsis, bacterial translocation to tissues or blood, and bacteremia, particularly in immunocompromised individuals (Suez et al. 2019).

For these reasons, instead of live microorganisms, the use of postbiotics is reported to be a more stable and safer alternative for industrial applications. Due to their higher pH and temperature stability (Toushik et al. 2023), postbiotics are more stable in foods than live microorganisms. They also provide technological advantages, including minimal interaction with the food matrix or its components, which contributes to an extended shelf life (de Almada et al. 2016). Furthermore, because they do not rely on live strains, the risk of microbial contamination is eliminated (Aguilar‐Toalá et al. 2018). Postbiotics also help avoid undesirable changes, such as excessive acidification after fermentation, and positively influence the sensory properties of the final products (de Almada et al. 2016). Additionally, some postbiotic‐containing products do not require cold chain storage or transportation, reducing logistical challenges (Shigwedha et al. 2014). Furthermore, they can be paired with packaging materials designed to minimize oxidative stress, enhancing product quality (Granato et al. 2010).

In addition to their technological advantages, the bioprotective properties of LAB strain‐derived postbiotics have been demonstrated in food products such as meat (Toushik et al. 2023) and dairy products (Hossain et al. 2021), emphasizing their potential use as alternative biopreservative agents.

Since postbiotics provide an effective means of enhancing functional and bioprotective properties in food products, this study evaluated the bioactivity and bioprotective potential of postbiotics derived from 10 indigenous LAB strains. Functional analyses included in vitro assessments of antimicrobial and antioxidant activities, along with measurements of phenolic and organic acid content. To investigate the bioprotective properties, postbiotics were incorporated into pasteurized milk, both with and without *Escherichia coli* . Microbiological characteristics were observed on days 1, 5, and 10 over the product's 10‐day shelf life.

## 2 Materials and Methods

### 2.1 Preparation of Bacterial Cultures

The ten lactic acid bacterial cultures used in this study were those isolated and characterized in an earlier study (Ozturkoglu Budak et al. 2016) and maintained in The Netherlands Culture Collection of Bacteria—NCCB (cultures 1–6) and in the Department of Dairy Technology, Ankara University (cultures 7–10), respectively. The individual cultures were as follows:

1. *Lactococcus* (*Lc*.) *lactis* subsp. *lactic* NCCB 100539
2. *Lactiplantibacillus* (*L*.) *paraplantarum* NCCB 100523
3. *Lactiplantibacillus* (*L*.) *plantarum* NCCB 100524
4. *Loigolactobacillus* (*L*.) *coryniformis* NCCB 100543
5. *Levilactobacillus* (*Lb*.) *brevis* NCCB 100526
6. *Enterococcus* (*E*.) *faecium* NCCB 100527
7. *Enterococcus* (*E*.) *faecalis* ZH‐2
8. *Streptococcus* (*St*.) *thermophilus* 1‐K_4_
9. *Lactobacillus* (*Lb*.) *helveticus* 9‐B_5_
10. *Lactobacillus* (*Lb*.) *bulgaricus* 23

During the activation of LAB strains, stock cultures (10^9^ CFU/mL) stored at −80°C were inoculated into MRS and M17 broth (Merck, Germany) depending on the strain type. For Lactobacilli and Lactiplantibacillus, MRS broth was used, and for Lactococci, Streptococci, and Enterococci, M17 broth was used. Lactobacilli strains were incubated at 37°C for 48 h under anaerobic conditions (Microbiology Anaerocult A, Merck). Lactococci were aerobically incubated for 24 h at 30°C, and streptococci and enterococci were incubated aerobically for 24 h at 37°C. To observe the bacterial growth pattern, optical density (OD) was checked using a spectrophotometer at 540 nm (Lambda 25 UV/Vis, PerkinElmer, Singapore) and taken for each strain at the beginning and every 2 h during the applied incubation periods mentioned before.

### 2.2 Obtaining Postbiotics

Both cell‐free extracts and cell surface/wall components were obtained as postbiotics. Cell‐free extracts were obtained at the end of the logarithmic phase, when bacterial metabolites were most abundant and the OD value was at its peak for each bacterium. These OD values were confirmed by plating to determine viable colony‐forming units (as counts), and a standard graph was prepared by plotting OD against cell counts. The grown cultures in broths after the incubation period were centrifuged at 8000 rpm for 5 min at 4°C to separate pellets and the cell‐free supernatants. The supernatants were kept at −80°C for further analysis. The pellet obtained by centrifugation was washed twice in 50 mM potassium phosphate buffer (pH 7.0) and re‐dispersed in sterile PBS (phosphate‐buffered saline) to obtain cell wall components. After the cell count in the dispersed suspension was fixed at 8 log cfu/mL, it was subjected to 4 sonication cycles at 300 W for 7 min using a probe sonicator (Bandelin, Sonopuls, Germany). A 2‐min ice‐holding period was applied between each cycle. Cell debris was separated by re‐centrifugation (10 000 x *g* at 4°C for 10 min) (Ashraf et al. 2014). After this process, cell viability was determined by the spread plate method, and no live cells were found. The obtained cell‐free extract and cell wall components were combined and subjected to functional and biopreservation analyses. Postbiotics were passed through membrane filters with a pore diameter of 0.22 μm (Millipore, Merck) to ensure the elimination of live cell presence before all analyses.

### 2.3 Functional Analysis of Postbiotics

#### 2.3.1 Determination of Phenolic Contents

During the phenolic content analysis, 100 μL of the sample, 500 μL of Folin–Ciocalteu reagent (Merck, Germany), 1.5 mL of 20% Na_2_CO_3_, and 7.9 mL of distilled water were mixed in a test tube. The mixtures were vortexed and kept in a dark place at room temperature for an incubation period of 1 h. After this duration, the absorbances were read in triplicate at 760 nm in a UV–Vis spectrophotometer (PerkinElmer, Lambda 25, USA). Calculations were done using by gallic acid standard curve. For this aim, 5 mg/mL gallic acid was prepared as the main stock solution, and the working solutions were prepared at the concentrations of 4, 3, 2, 1, 0.75, 0.5, 0.25, 0.10, 0.05 mg/mL. The values were expressed in mg GAE/mL (*R* ^2^: 0.97). MRS and M17 media alone were used as controls in the analysis, depending on the LAB strain.

#### 2.3.2 Determination of Antioxidant Activity

##### 2.3.2.1 DPPH Radical Scavenging Capacity

To perform antioxidant analyses via DPPH (2,2‐Diphenyl‐1‐picrylhydrazyl, Sigma‐Aldrich, MA, USA) assay, 100 μL of sample was added to 2 mL of DPPH solution (0.1 mM DPPH prepared in MeOH). All samples were kept in the dark and incubated for 30 min at 4°C. After 30 min, the absorbance of all samples was checked in the spectrometer (PerkinElmer, Lambda 25, USA) at 517 nm wavelength. The inhibition % was estimated by using the following formula:

$$\%\text{Inhibition}=\frac{A_{\text{Blank}-}-A_{\text{Sample}}}{A_{\text{Blank}}}\times 100$$

*A*_blank_: Absorbance of DPPH solution with water instead of the sample. *A*_sample_: Absorbance of DPPH solution with the sample.

Antioxidant activity of each postbiotic was calculated by using inhibition % values according to the Trolox standard curve that was plotted by using the Trolox concentrations of 5, 2.5, 1, 0.25, 0.1, and 0.05 mM. The values were calculated in mM TEAC (Trolox Equivalent Antioxidant Capacity) with an *R* ^2^ of 0.98. MRS and M17 media alone were used as controls in the analysis, depending on the LAB strain.

##### 2.3.2.2 ABTS Radical Scavenging Capacity

ABTS (2,2′‐azinobis (3‐ethylbenzothiazoline‐6‐sulfonic acid), Sigma‐Aldrich, MA, USA) was prepared in deionized water at a concentration of 7 mM. This solution, containing 2.45 mM potassium persulfate, was kept in a dark room for 12–16 h to produce sufficient amounts of free radicals. Subsequently, the obtained solution was diluted with 5 mM phosphate‐buffered saline solution (pH 7.4), and 10 μL, 20 μL, and 30 μL postbiotic samples were individually added to 1 mL of this dilution to measure the absorbances at 734 nm on a spectrophotometer. Percent radical removal values were determined by applying linear regression analysis by plotting them against sample amounts. The decrease in absorbance was calculated as TEAC. Trolox standards were prepared in amounts with final concentrations varying between 5–20 μM, and the % radical scavenging activity and TEAC value were calculated as follows (Rana et al. 2018).

$$\%\text{Radical scavenging activity}=\frac{A_{\text{Control}-}A_{\text{Sample}}}{A_{\text{Control}}}\times 100$$

*A*_sample_: absorbance obtained by co‐measurement of the sample with ABTS working solution. *A*_control_: absorbance obtained by measuring the ABTS working solution together with water instead of the sample.

$$\begin{array}{c} \text{TEAC}\;\left(\mathrm{mM}\;\text{trolox}/\mathrm{mL}\;\text{sample}\right)\\ =\frac{\text{Slope of the radical scavenging activity curve of the sample}}{\text{Slope of the Trolox standard curve}}\times \mathrm{DF} \end{array}$$

DF, dilution factor.

#### 2.3.3 Antimicrobial Analyses

The antimicrobial properties of postbiotics were tested against 6 different pathogens *Staphylococcus aureus* (ATCC 25923), *Mycobacterium tuberculosis* (ATCC 27294), *Escherichia coli* (ATCC 25922), *Bacillus cereus* (ATCC 14579), *Salmonella enterocolitica* (ATCC 14028), *Enterococcus faecalis* (ATCC 29212) via the agar well diffusion method. The selective medium of Brain Heart Infusion (BHI, Merck, Germany) was used for the analyses. The stock cultures of each pathogen (~10^8^ log CFU/mL) were activated in BHI broth individually, and incubation was performed at 37°C for 20 h. Wells were formed on BHI agar with a sterile cork‐borer. During analyses, for each plate, 100 μL of activated pathogen was spread onto the plate from the diluted concentration of 10^6^ CFU/mL, and 50 μL of postbiotic obtained from each LAB was introduced individually into the designated well. On each plate, one well was prepared with ampicillin (Merck, 0.2% prepared in Ethanol) as a positive control, and another well contained either MRS or M17 medium (Merck, Germany), depending on the strain, as a negative control. The plates were incubated at 37°C for 24 h. The antimicrobial activity was observed by measuring the clear zone around each well.

#### 2.3.4 Organic Acid Profile

The organic acids present in each postbiotic were determined via an HPLC system (1100 series; Agilent Technology, CA, USA) as described by Bulat and Topcu (2020). 0.013 N H_2_SO_4_ (Supelco, St. Louis, USA) was used as the mobile phase. MetaCarb 87H column (300 × 7.8 mm) (Agilent Technologies, CA, USA) was used with the temperature of 65°C and the flow rate of 0.8 mL/min. Organic acids were detected at a wavelength of 210 nm. Samples were prepared by adding 5 mL of the individual postbiotic to 1 mL of 0.013 N H_2_SO_4_. The mixtures were vortexed for 2 min, passed through a 0.22 μm syringe filter (Isolab, Germany), and a 20 μL sample was injected into the HPLC. Standard calibration curves were plotted for each organic acid at the concentrations of 1600, 800, 400, 200, 100, and 50 ppm. MRS and M17 media alone were used as controls in the analysis, depending on the LAB strain.

#### 2.3.5 Biopreservation Potential of Postbiotics in Pasteurized Milk

**FIGURE 1:**
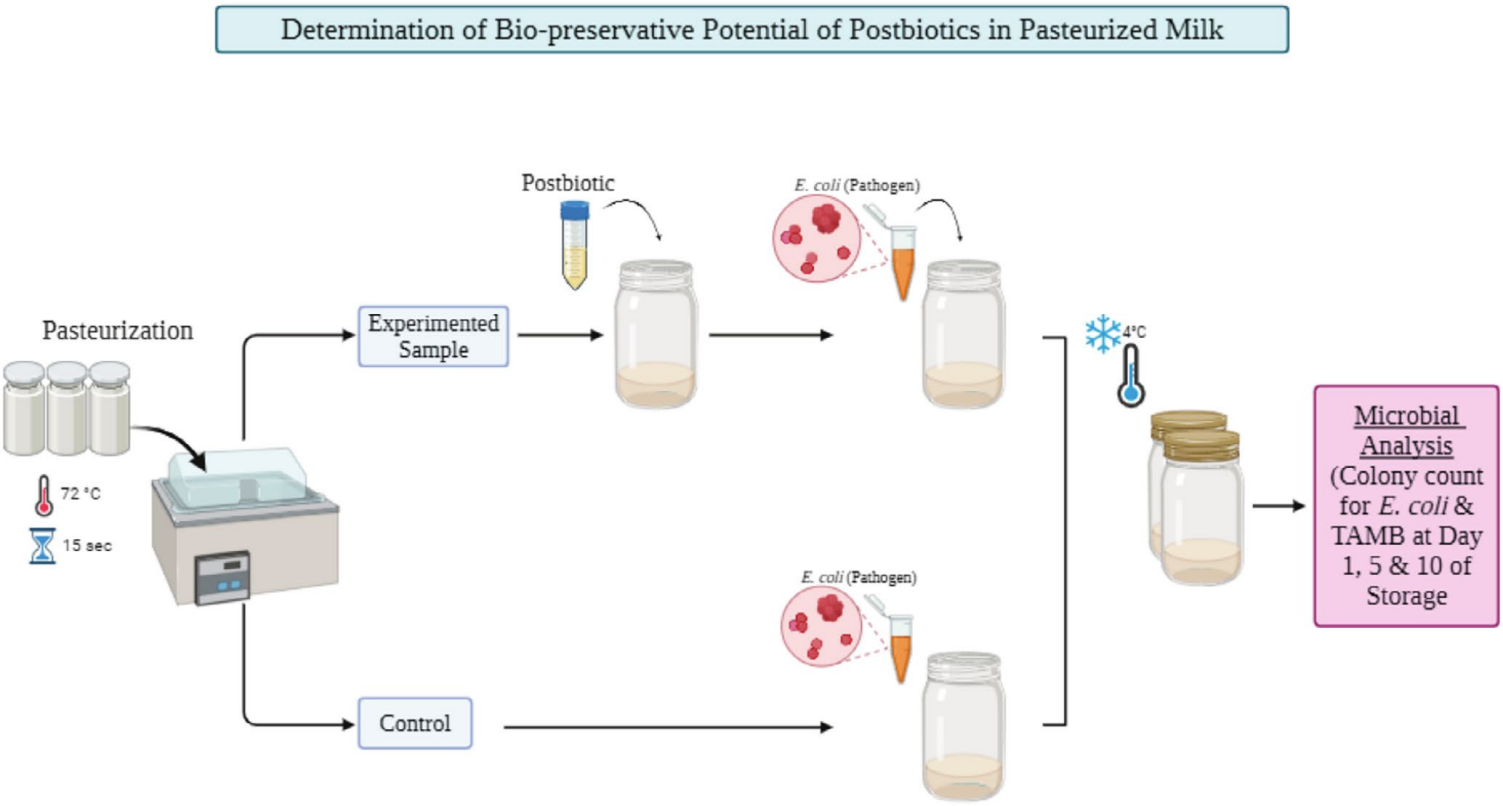
Experimental design for the bio‐preservation analyses.

*Escherichia coli* (ATCC 25922) was selected as the target pathogen for determining the biopreservation potential in pasteurized milk because, during in vitro antimicrobial analyses, the majority of the tested postbiotics exhibited antimicrobial activity against this strain compared to the other five pathogens. 1 mL of postbiotic belongs to each LAB, and 100 μL *E. coli* (10^8^ CFU/mL) was incorporated into 100 mL of pasteurized milk (72°C for 2 min) and stirred gently for a homogeneous mixture. Pasteurized milk with only *E. coli* and without any postbiotics, and *E. coli was* also prepared for comparing the results (Figure 1). The samples were stored at 4°C for 10 days. The count of *E. coli* and Total Aerobic Mesophilic Bacteria (TAMB) was analyzed on the SMAC Agar (with CT Supplement) and Plate Count Agar (PCA), respectively, by using the pour plate method on days 1, 5, and 10 of the storage period. Plates were incubated at 37°C for 24 h for both bacterial groups. All media were obtained from Merck (Germany).

### 2.4 Statistical Analysis

Statistical analyses were carried out using the Minitab package program (Software version 17, State College, USA). Analysis of variance (ANOVA) was performed to determine statistical differences among samples. Significant differences among the storage days of pasteurized milk were measured using Tukey's Multiple Range Test (*p* ≤ 0.05) at a 95% confidence level.

## 3 Results and Discussion

### 3.1 Functional Properties of Postbiotics

#### 3.1.1 Phenolic Contents

The direct phenolic content of each postbiotic was calculated by subtracting the phenolic content of the control (MRS or M17 media) from the phenolic content of the respective postbiotic. Based on the obtained data, postbiotics from *L. paraplantarum* NCCB 100523 (0.71 mg GAE/mL) exhibited the highest phenolic content. Following this strain, *E. faecalis* ZH‐2 and *L. plantarum* NCCB 100524 showed moderate levels of phenolic content (0.65 mg GAE/mL) in their postbiotics. *L. plantarum* NCCB 100524 was also found to be the highest phenolic content producer (Table 1). Wu et al. (2021) reported that *L. plantarum* exhibits a high capacity to metabolize phenolic and organic acids. This enhanced metabolic ability in samples fermented by *L. plantarum* led to a significant increase in phenolic content and antioxidant activity. Our findings showed similarity to the observations of Li et al. (2021), who also confirmed the production of high amounts of phenolic compounds by LAB species of *L. plantarum,* as 2 g GAE/L. Grigore‐Gurgu et al. (2024) also confirmed the production of high amounts of phenolic contents by *L. paraplantarum* MIUG BL74 (Lpb 74) and *L. plantarum* MIUG BL21 (Lpb 21) under different medium conditions. The main reason for the high amount of phenolic content lies in the ability of LAB to adapt to the native environment (Rodríguez et al. 2009). Liu et al. (2021) also suggested that high phenolic content could be contributed by the presence of hydrolytic enzymes that are secreted by LAB during incubation or might be released by deglycosylation of glycosylated phenols. The high amount of phenolic contents detected in postbiotics of *L. plantarum* NCCB 100524 could also be directly linked with β‐glucosidase enzyme, which is present in the metabolites of *L. plantarum* , which hydrolyzes the flavonoid compounds and increases the bioavailability of phenolic compounds (Degrain et al. 2020). Other than *L. plantarum* NCCB 100524, fermentation by *E. faecalis* M2 significantly increased the phenolic acid content in wheat bran, demonstrating a notable enhancement of its phenolic properties (Mao et al. 2020). A similar outcome was observed with blueberry juice, where the phenolic content increased following fermentation with *Lc. lactis* (Gao et al. 2022). According to Zhou et al. (2023), the *L. paraplantarum* LS‐5 strain was able to produce significant amounts of polyphenols and flavonoids. Higher levels of phenolic compounds were also observed in the postbiotics of *Lb. brevis* NCCB 100526 compared to the findings of Sornsenee et al. (2021), where only > 0.25 mg GAE/mL was reported. The results for the phenolic contents obtained from *Lb. bulgaricus* 23 were similar to the results observed by Hamad et al. (2022).

**TABLE 1:** Functional properties of postbiotics.

Sample	Antioxidant capacity		Phenolic content (mg GAE/mL)
	DPPH (TEAC mM Trolox)	ABTS (mM TEAC/mL)	
*E. faecalis* ZH‐2	3.60 ± 0.11^a^	4.15 ± 0.22^a^	0.66 ± 0.04^ab^
*E. faecium* NCCB 100527	3.46 ± 0.16^a^	3.21 ± 0.17^b^	0.63 ± 0.01^b^
*St. thermophilus* 1‐K_4_	3.10 ± 0.07^ab^	2.80 ± 0.25^b^	0.55 ± 0.00^b^
*Lc. lactis* NCCB 100539	3.61 ± 0.18^a^	4.13 ± 0.09^a^	0.64 ± 0.06^ab^
*L. plantarum* NCCB 100524	3.70 ± 0.25^a^	3.45 ± 0.31^ab^	0.66 ± 0.03^ab^
*L. paraplantarum* NCCB 100523	3.69 ± 0.06^a^	3.36 ± 0.13^ab^	0.72 ± 0.03^a^
*L. coryniformis* NCCB 100543	2.23 ± 0.33^b^	1.90 ± 0.28^c^	0.54 ± 0.08^ab^
*Lb. brevis* NCCB 100526	3.24 ± 0.02^ab^	2.89 ± 0.21^bc^	0.55 ± 0.06^b^
*Lb. helveticus* 9‐B_5_	1.88 ± 0.00^bc^	2.02 ± 0.40^c^	0.56 ± 0.00^ab^
*Lb. bulgaricus* 23	2.35 ± 0.46^b^	2.48 ± 0.32^bc^	0.60 ± 0.00^ab^
Control MRS media	1.80 ± 0.09^bc^	1.88 ± 0.24^c^	0.009 ± 0.00^c^
Control M17 media	1.25 ± 0.08^c^	1.33 ± 0.11^c^	0.010 ± 0.00^c^

*Note:* The mean values with different lower‐case letters in the same column are significantly different (*p <* 0.05).

#### 3.1.2 Antioxidant Activities

Antioxidant activities of postbiotics from LAB species are given in Table 1. The measurement values of the corresponding control media (MRS or M17) were subtracted from those of each postbiotic sample, revealing the direct antioxidant effect of the bacterial strain's postbiotic. According to the obtained results, postbiotics from *Lc. lactis* NCCB 100539, *E. faecalis* ZH‐2, *E. faecium* NCCB 100527, *L. plantarum* NCCB 100524, and *L. paraplantarum* NCCB 100523 showed the highest antioxidant activities among all the tested postbiotics, while the lowest antioxidant activities were determined from the postbiotics of *L. coryniformis* NCCB 100543, *Lb. bulgaricus* 23, and *Lb. helveticus* 9‐B_5_. The antioxidant capacities of most postbiotics measured by ABTS and DPPH assays showed similar patterns. Antioxidant activity of postbiotics from *L. plantarum* NCCB 100524 was much higher compared to the findings of Noori et al. (2023). Similarly, high antioxidant properties of postbiotics were observed from *L. plantarum* NJAU‐01 (Liu et al. 2021), *L. plantarum* strain Lp‐790, and Lp‐998 (Zago et al. 2017) in different studies. In a similar way, we can observe the higher DPPH activity of postbiotics of *L. plantarum* ATCC 14917 and *Lb. brevis* ATCC 8287 from the findings of Vougiouklaki et al. (2023). Here, the main reason lies in the time duration that was initially provided for incubation. The average incubation time applied was 20 h and 24 h for *Lb. plantarum* NCCB 100524 and *Lb. brevis* NCCB 100526, respectively, while in the above‐mentioned study, the incubation time was extremely high of around 5 days. Due to the high capacity of *L. plantarum* for metabolizing phenolic and organic acids, its enhanced metabolic ability significantly increased the antioxidant activity in fermented blackberry and blueberry juices (Wu et al. 2021). High phenolic content detected from the postbiotics of *E. faecalis* ZH‐2 could be responsible for showing high antioxidant activities, similar to *L. plantarum* NCCB 100524. Enzymes such as catalase, peroxide dismutase, and glutathione peroxidase are considered antioxidant microbial enzymes (Aghebati‐Maleki et al. 2021). These enzymes suppress reactive oxygen species. In addition, it has been emphasized in the literature that peptides in postbiotics have antioxidant properties (Blazheva et al. 2022). It has been reported that organic acids such as lactic acid and acetic acid in postbiotics have radical binding activity due to their hydroxyl group content and are good electron donors, and antioxidant activity has also been associated with exopolysaccharides, lipoteichoic acid, and cell surface proteins (Izuddin et al. 2018). Postbiotics of *St. thermophilus* in our findings also have a similar radical scavenging activity with the results of Wang and Li (2022). Additionally, *Lc. lactis* NCCB 100539 also showed comparatively higher antioxidant activity of 3.58; another strain belonging to *Lc. lactis* was also reported in a similar amount by De Marco et al. (2018). Some differences observed in comparison with the literature suggest that the antioxidant activity of postbiotics may vary depending on factors such as the microbial strain, the type of matrix used, the fermentation duration, and the specific fraction analyzed (cellular or cell‐free).

In terms of postbiotics taken from *Lb. bulgaricus* 23, the antioxidant activity was not significantly high as compared to that of other postbiotics. The results for the postbiotics of *Lb. bulgaricus* 23 were contradictory to the ones obtained by Liu and Pan (2010), who observed higher antioxidant potential of postbiotics of *Lb. bulgaricus* BCRC 10696. The reason could be the method that was employed to obtain these postbiotics, as in the above‐mentioned study, the heat‐killed cells (121°C) and their cytoplasmic cell fractions were taken as postbiotics instead of metabolites secreted by bacterial cells. In the case of postbiotics of *Lb. helveticus* 9‐B_5_ and *Lb. bulgaricus* 23, although they possessed a good amount of phenolic contents, these contents did not exhibit good antioxidant activities.

#### 3.1.3 Antimicrobial Activities

All postbiotics exhibited an inhibition zone of less than 10 mm, indicating very low antimicrobial activity. However, when the effectiveness of each postbiotic was compared across different pathogens, all demonstrated measurable inhibition against *E. coli* , with zones of at least 7 mm in diameter around the well. This observation may offer insight into the potential antimicrobial activity that could be achieved by increasing the concentration of postbiotics in the product. The control (MRS or M17 media alone) showed no inhibitory effect against the pathogens tested. Postbiotics from *L. plantarum* NCCB 100524, *L. paraplantarum* NCCB 100523, *Lc. lactis* NCCB 100539, *St. thermophilus* 1‐K_4_, and *E. faecalis* ZH‐2 showed similar antimicrobial activity against *E. coli* (8 mm inhibition zone). This was also observed by using the CFS of *Lb. plantarum* strain isolated from kefir (Puertollano et al. 2009). In general, postbiotics derived from *Lactobacillus* species were found to be effective primarily against Gram‐negative bacteria, whereas those produced by *Enterococcus* and *Streptococcus* species also demonstrated activity against Gram‐positive bacteria. Among *Lactobacillus* species, only *L. paraplantarum* NCCB 100523 and *Lc. lactis* NCCB 100539 was found to be effective against Gram‐positive pathogens. In contrast, *St. thermophilus* 1‐K_4_, *E. faecium* NCCB 100527, and *E. faecalis* ZH‐2 exhibited inhibitory effects, particularly against *M. paratuberculosis* and *B. cereus* , both of which are Gram‐positive bacteria.

The antibacterial effects of postbiotics from LAB have been widely reported. In particular, postbiotic compounds obtained from species such as *L. plantarum* , *L. helveticus* , *L. casei* , *L. rhamnosus*, and *L. fermentum* are effective against both food pathogens and opportunistic microorganisms (Abbasi et al. 2024; Aghebati‐Maleki et al. 2021). In our study, the inhibitory effect of postbiotics obtained from *L. paraplantarum* NCCB 100523, *L. plantarum* NCCB 100524, *E. faecalis* ZH‐2, *S. thermophilus* 1‐K_4_, *Lc. lactis* NCCB 100539, and *Lb. helveticus* 9‐B_5_ is much more potent compared to the other postbiotics (showing 8 to 10 mm inhibition zone), which is likely attributable to the synergistic effect of metabolite diversity and the high levels of bioactive postbiotic components synthesized by these strains. This comparison will be important in determining the concentration of the relevant postbiotics when they are intended to be integrated into a product. Upon examining the antioxidant activity and phenolic content of the postbiotics derived from these strains, it is evident that *L. plantarum* NCCB 100524, *L. paraplantarum* NCCB 100523, *E. faecalis* ZH‐2, *E. faecium* NCCB 100527, *L. brevis* NCCB 100526, and *Lc. lactis* NCCB 100539, all of which exhibit strong antimicrobial activity, also produce high levels of antioxidants and phenolic compounds.

When evaluating the antimicrobial activity of postbiotics about their organic acid content, *L. helveticus* 9‐B_5_ emerges as the primary producer of acetic acid, while *S. thermophilus* 1‐K_4_ and *E. faecium* NCCB 100527 are the main formic acid producers. *E. faecium* NCCB 100527 was also found to be a potential producer of acetic acid. This is thought to be the reason for their high antimicrobial activity. Similarly, Abbasi et al. (2024) reported that postbiotics derived from *L. helveticus* strains exhibited antibacterial activity against *S. aureus* and *E. coli* O157:H7. Additionally, the inhibition of spore‐forming pathogens such as *B. cereus* and enterotoxin‐producing pathogens such as *Y. enterocolitica* by these postbiotics suggests a promising potential for their application in enhancing food safety. Similarly, Toy et al. (2015) demonstrated that postbiotics produced by *S. thermophilus* were effective against *S. aureus* .

The antimicrobial activity of the cell‐free supernatants from *L. plantarum* NCCB 100524 and *Lb. brevis* NCCB 100526 against *E. coli* was also observed in the findings of Yolmeh et al. (2017). However, in their study, a mixture of postbiotics was used against *E. coli* , rather than assessing the individual antimicrobial effects of single postbiotics. In contrast, no antimicrobial activity was detected from any postbiotic against *B. cereus* and *S. aureus* . The low antimicrobial activity against *S. aureus* was contrary to the findings of Taj et al. (2022), who observed adequate antimicrobial activity against *S. aureus* by various *St. thermophilus* strains. This could be possible due to some variations among different *S. aureus* strains. Additionally, neither *St. thermophilus* nor *Lc. lactis* NCCB 100539 demonstrated any antimicrobial effect against *S. aureus* (Hladíková et al. 2012). In another study, the postbiotics of *Lb. plantarum* NRRL B‐4496 strain showed high antimicrobial activity against *S. aureus* (ATCC 25923), which was due to the secretion of different amounts of organic acids by strains of the same bacteria (Arrioja‐Bretón et al. 2020).

**FIGURE 2:**
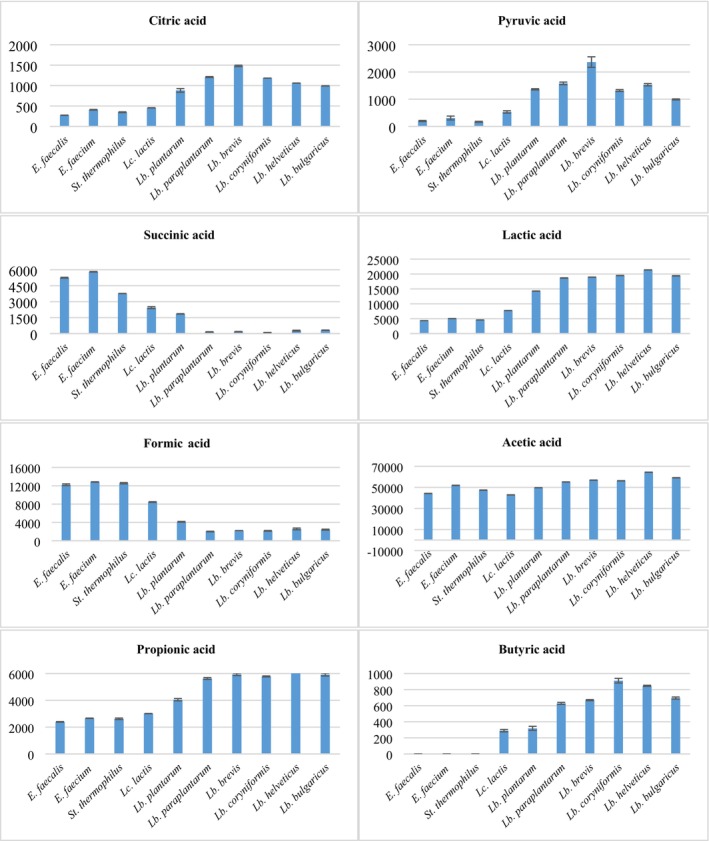
Organic acid amounts (μg/g) of postbiotics.

Postbiotics from *E. faecalis* ZH‐2 and *Lc. lactis* NCCB 100539 was significantly effective against *M. tuberculosis* , which was shown by the 10 mm diameter inhibition zone. Additionally, *S. enterocolitica* was inhibited by the postbiotics of *L. plantarum* NCCB 100524, *L. paraplantarum* NCCB 100523, *Lb. helveticus* 9‐B_5_, and *L. coryniformis* NCCB 100543, showing a zone diameter of 8 to 9 mm. Regarding the postbiotics of *Lb. brevis* NCCB 100526, some antimicrobial activity was observed (8 mm inhibition zone) against *E. coli*, which is believed to be associated with the high levels of citric acid and pyruvic acid detected in these postbiotics (Figure 2). Citric acid creates an acidic environment when added to certain food products, thereby inhibiting the survival of many pathogenic microorganisms (Hosseini et al. 2021). In contrast, Arrioja‐Bretón et al. (2020) reported no inhibition zone against *E. coli* when using the cell‐free supernatant of *Lb. brevis* NCCB 100526. This discrepancy is likely due to differences in the strains of *Lb. brevis* was used to obtain the postbiotic supernatants. In this context, based on the low inhibitory effect of specific postbiotics against certain pathogens, preliminary data on the concentrations required for an effective antimicrobial effect in product integration are presented.

#### 3.1.4 Organic Acid Amounts

The amounts of organic acids (μg/g) identified in the postbiotics are presented in Figure 2. The direct organic acid amounts were calculated by subtracting the control value (MRS or M17 media) from the value of the respective postbiotic. In terms of citric acid, *Lb. brevis* NCCB 100526 showed the highest, and *E. faecalis* ZH‐2 showed the lowest citric acid quantity at 1482.31 and 272.68 μg/g, respectively. Another low citric acid‐producer strain has been identified as *St. thermophilus* 1‐K_4_. The amount of citric acid produced by LAB depends on the source and amount of sugar present in the growth medium, and lower amounts of lactose in M17 might cause low production of citric acid (Hossain et al. 1984). In a study by Li et al. (2021), similar amounts of citric acid were observed for *Lb. helveticus* and *L. plantarum* when inoculated in jujube juice and incubated for 48 h at 37°C.

Pyruvic acid is an ɑ‐keto acid which is considered an important intermediate metabolite produced during the breakdown of carbohydrates by LAB. Significantly high amounts of pyruvic acid were detected in the postbiotics of *Lb. brevis* NCCB 100526 (2363.5 μg/g), followed by postbiotics of *L. paraplantarum* NCCB 100523 (1581.7 μg/g). Postbiotics from *St. thermophilus* 1‐K_4_ showed the lowest pyruvic acid amount of around 167.7 μg/g. High pyruvic acid production by *Lb. brevis* NCCB 100526 could be due to the presence of pyruvic acid kinase activity, which is often secreted by *Lb. brevis* and causes the conversion of 3‐phosphoglyceric acid to pyruvate (Eltz and Vandemark 1960). Some *Streptococcus* strains produce pyruvic acid as an intermediate acid, which is either oxidized to acetic acid or reduced to lactic acid. Hence, the decreased amount of pyruvic acid detected by *St. thermophilus* 1‐K_4_ could be due to its conversion into other organic acids.

Succinic acid has an inhibitory effect similar to acetic acid and could be effectively used as a biopreservative agent by acting as a buffer (Purohit and Mohan 2019). In this study, *Enterococcus* species produce the highest amount of succinic acid, with *E. faecium* NCCB 100527 producing 5808.6 μg/g and *E. faecalis* ZH‐2 producing 5249.4 μg/g. The variation in the composition of the growth medium plays a complex role in determining the amount of succinate and succinic acid produced by *Enterococcus* species because the succinic acid production is largely enhanced by the presence of yeast extract in M17 broth medium (Kang et al. 2000). The amount of succinic acid produced by *St. thermophilus* 1‐K_4_ and *Lc. lactis* NCCB 100539 was much higher than the findings of Özcelik et al. (2016), who revealed half the amount of succinic acid produced by *St. thermophilus* NCFB2392 in MRS broth but higher amounts in anchovy infusion broth, which could occur due to differences in bacterial strains and their performance across various growth media. *Lactobacillus* species, including *Lb. brevis* NCCB 100526, *L. paraplantarum* NCCB 100523, and *L. coryniformis* NCCB 100543, produced the lowest amounts of succinic acid. *Lactobacillus* produces succinic acid by utilizing diammonium citrate present in the MRS media. The ability to utilize diammonium citrate is linked to the bacterial strain, as not all *Lactobacillus* strains can produce succinic acid (Kaneuchi et al. 1988). The results for the postbiotics of all *Lactobacillu*s species show that these species have a minimum ability to produce succinic acid. The same lack of ability to produce succinic acid compared to other organic acids was also observed in the postbiotics of *Lb. helveticus* H9 (Rozhkova et al. 2023). Similarly, in another study by Jamaran et al. (2021), the lack of ability to produce succinic acid was also observed in the postbiotics of *Lb. reuteri*.

In terms of lactic acid, *Lactobacillus* species are found to produce comparatively high amounts. *Lb. helveticus* 9‐B_5_ produced the highest amount of 21368.3 μg/g, followed by that of *L. coryniformis* NCCB 100543 and *Lb. bulgaricus* 23. The results observed for the postbiotics of *Lb. helveticus* 9‐B_5_ were contrary to the findings of Rozhkova et al. (2023), who could not observe any lactic acid production by *Lb. helveticus* H9. This could be due to the difference in the characteristics of various strains of the same bacteria. Evivie et al. (2019) also reported that organic acid production is both strain‐ and species‐specific. Furthermore, the composition of the growth medium plays a crucial role in organic acid production. However, the results obtained for the postbiotics of *Lb. helveticus* 9‐B5 aligns with the findings of Jamaran et al. (2021), where the postbiotics from *Lb. reuteri* (a related genus) were found to be higher compared to others. Differences in the incubation time and glucose quantity could be a contributing factor to the decreased production of lactic acid. LAB species like *St. thermophilus* 1‐K_4_, *Lc. lactis* NCCB 100539 and *Enterococcus* species produced lower amounts of lactic acid. This might be because of further metabolism of lactic acid due to the formation of cytochromes by hemes, as stated by König and Fröhlich (2017).

*Enterococcus faecium* NCCB 100527 and *E. faecalis* ZH‐2 produced the highest amounts of formic acid at 12835.1 and 12232.20 μg/g, respectively. Significantly larger amounts of 12561.8 μg/g were also observed for *St. thermophilus* 1‐K_4_. Postbiotics produced by *Lactobacillus* species contained much lower concentrations of formic acid. However, the presence of formic acid in the growth medium supports the growth of LAB, which in turn leads to a further increase in formic acid production (Lei et al. 2024). Formic acid is produced in high amounts from pyruvate via the pyruvate formate‐lyase (PFL) pathway, in substrates having galactose instead of glucose. Since the MRS broth used for the incubation of *Lactobacillus* consists of glucose as the main sugar instead of galactose so it is thought that the activity of the PFL enzyme was carried out inefficiently (Melchiorsen et al. 2002). According to Noureldein et al. (2020), it was also observed that the amounts of formic acid produced by *Lb. brevis* were not as high as compared of other acids produced by the same bacteria. This could be due to the conversion of formic acid into other compounds.

All postbiotics showed almost similar amounts of acetic acid. The highest amount was detected in postbiotics from *Lb. helveticus* 9‐B_5_, followed by those produced by *Lb. bulgaricus* 23. The results for the postbiotics of *Lb. helveticus* 9‐B_5_ are similar to the results obtained by Rozhkova et al. (2023), who also showed higher acetic acid production in the growth medium of MRS broth. The production of acetic acid in MRS could be higher due to the presence of glucose used as an energy source by bacteria. An adequate amount of acetic acid produced by *L. plantarum* could be attributed to the activation of the phosphogluconate pathway and the intracellular route of the acetate kinase enzyme, which could contribute to the production of acetic acid, as explained by Filannino et al. (2014). Contrary to our findings, Özcelik et al. (2016) detected the amount of acetic acid near zero in the organic acid profile of *L. plantarum* , *Lc. lactis*, and *St. thermophilus*.

Mainly, *Lactobacillus* species were observed to produce higher amounts of propionic acid compared to *Enterococcus*, *Lactococcus*, and *Streptococcus*. The best propionic acid producer was determined as *Lb. helveticus* 9‐B_5_ (6379.1 μg/g). This high production of propionic acid by *Lb. helveticus* 9‐B_5_ is comparable to that of the propionic acid produced by *Lb. fermentum* IFO 3956, as shown by Behbahani et al. (2023).

Butyric acid was not detected in the postbiotics of both *Enterococcus* and *Streptococcus* species. However, all the *Lactobacillus* and *Lactococcus* species were found to be butyric acid producers, although in low concentrations. Among all LAB, the highest quantities were determined in the postbiotics of *L. coryniformis* NCCB 100543 (910.41 μg/g) and *Lb. helveticus* 9‐B_5_ (846.5 μg/g), respectively. Some amount of butyric acid was also detected in the postbiotic of *L. plantarum* NCCB 100524. The production of butyric acid by *L. plantarum* and *Lb. brevis* was also observed by Kam et al. (2011), but it was in small amounts. There is an important link between the production of butyric acid and the presence of yeast extract in MRS broth, which positively contributes to the availability of biomass for bacterial strains (Aiello et al. 2023). Nazzaro et al. (2012) also highlight the positive effect of yeast extract as a carbon source, which helps stimulate LAB to produce butyric acid.

### 3.2 Microbial Analysis of Pasteurized Milk Integrated With Postbiotics and Pathogens

The antimicrobial properties of postbiotics are primarily attributed to the presence of bacteriocins, organic acids, and other minor metabolites. These compounds are known to inhibit the growth of pathogenic microorganisms. Based on the organic acid concentrations presented in Figure 2, the sharp reduction in pathogen counts observed with *E. faecium* NCCB 100527 may be linked to its high production of formic and acetic acids. Similar to our findings, Mancini et al. (2025) also reported *E. faecium* as an effective dairy bio‐preservative agent.

Table 2 shows the count of *E. coli* and TAMB on days 1, 5, and 10 of the storage period. Based on the *E. coli* count, a sharp decline from 6.05 log CFU/mL at day 1 to 2.5 log CFU/mL at day 10 was observed in samples containing postbiotics of *E. faecium* NCCB 100527. Postbiotics obtained from *Lc. lactis* NCCB 100539 also impacted the pathogen population, with a log reduction observed from 5.99 log CFU/mL on day 1 to 5.30 log CFU/mL on day 10. Similar antimicrobial activity by CFS obtained from another strain of *Lc. lactis* (ATCC‐11454) was also observed by Millette et al. (2004). However, the sample having postbiotic from *St. thermophilus* 1‐K_4_ showed a limited decrease in pathogen colony until day 5 and no decrease after that day. Limited antimicrobial activity was also shown by some other strains of *St. thermophilus* (Taj et al. 2022). Among *Lactobacillus* species, although all samples demonstrated a decrease in *E. coli* counts after 10 days of storage, samples containing postbiotics from *Lb. brevis* NCCB 100526, *Lb. helveticus* 9‐B_5_, and *Lb. bulgaricus* 23 showed significantly greater reductions in pathogen counts during the storage period (*p* < 0.05). The activity of the postbiotics from *Lb. brevis* NCCB 100526 could be comparable to the results obtained by Barzegar et al. (2023), but since a much higher percentage of postbiotics was used, the antimicrobial effect obtained was higher than our findings. In the samples containing postbiotics from *Lb. helveticus* 9‐B_5_ and *Lb. bulgaricus* 23, a decrease in pathogen count was observed, dropping from 5.96 to 4.99 and from 6.01 to 5.35 log CFU/mL, respectively, by the final day of storage. These species were identified as the main producers of acetic acid, which is believed to be the reason for their inhibitory effect against pathogens. A slight reduction was also observed in samples containing postbiotics from *L. paraplantarum* NCCB 100523, with pathogen counts decreasing from 5.92 to 5.35 log CFU/mL. This strain was also identified as a major producer of formic acid (Figure 2). The initial composition of the growth medium for *L. plantarum* could affect its antimicrobial activity. The addition of 5% glycerol in the MRS broth could significantly affect the production of bacteriocin by *L. plantarum* Cys5‐4, which results in an increased amount of antimicrobial activity (Tenea and Barrigas 2018). Bleicher et al. (2010) identified these bacteriocins and observed their inhibition spectrum against gram‐negative *E. coli* bacteria.

**TABLE 2:** *Escherichia coli* and TAMB count (log CFU/mL) of pasteurized milks during storage.

Sample	Day	*E. coli* count	TAMB count
Positive control ( *E. coli* )	1	5.76 ± 0.00^aA^	6.82 ± 0.03^A^
	5	6.65 ± 0.00^aAB^	6.84 ± 0.14^B^
	10	6.81 ± 0.21^aB^	6.70 ± 0.00^B^
Negative control (PS)	1	0.00	0.95 ± 0.08^A^
	5	0.00	1.11 ± 0.19^AB^
	10	0.00	1.33 ± 0.21^B^
*E. faecalis* ZH‐2	1	5.45 ± 0.64^abA^	6.36 ± 0.18^A^
	5	5.20 ± 0.28^abAB^	5.86 ± 0.78^B^
	10	5.15 ± 0.21^abB^	6.29 ± 0.02^B^
*E. faecium* NCCB 100527	1	6.05 ± 0.20^bA^	6.59 ± 0.15^A^
	5	5.57 ± 0.81^bAB^	5.94 ± 0.33^B^
	10	2.50 ± 3.54^bB^	5.98 ± 0.54^B^
*St. thermophilus* 1‐K_4_	1	5.80 ± 0.14^abA^	6.39 ± 0.54^A^
	5	5.35 ± 0.07^abAB^	5.94 ± 0.65^B^
	10	5.35 ± 0.49^abAB^	5.63 ± 0.46^B^
*Lc. lactis* NCCB 100539	1	5.99 ± 0.01^abA^	6.45 ± 0.08^A^
	5	5.28 ± 0.03^abAB^	6.08 ± 0.54^B^
	10	5.30 ± 0.43^abB^	5.94 ± 0.09^B^
*L. plantarum* NCCB 100524	1	5.91 ± 0.21^abA^	6.25 ± 0.11^A^
	5	6.17 ± 0.05^abAB^	5.99 ± 0.30^B^
	10	5.81 ± 0.43^abB^	6.56 ± 0.30^B^
*L. paraplantarum* NCCB 100523	1	5.92 ± 0.34^abA^	6.49 ± 0.23^A^
	5	6.00 ± 0.37^abAB^	6.00 ± 0.73^B^
	10	5.35 ± 0.49^abB^	5.78 ± 0.25^B^
*L. coryniformis* NCCB 100543	1	5.57 ± 0.81^abA^	6.44 ± 0.33^A^
	5	5.30 ± 0.43^abAB^	5.98 ± 0.19^B^
	10	5.00 ± 0.00^abB^	5.70 ± 0.49^B^
*Lb. brevis* NCCB 100526	1	5.97 ± 0.24^abA^	6.48 ± 0.67^A^
	5	5.59 ± 0.83^abAB^	6.18 ± 0.04^B^
	10	5.00 ± 0.00^abB^	5.73 ± 0.07^B^
*Lb. helveticus* 9‐B_5_	1	5.96 ± 0.51^abA^	6.41 ± 0.58^A^
	5	5.30 ± 0.43^abAB^	5.74 ± 0.37^B^
	10	4.99 ± 0.69^abB^	5.74 ± 0.37^B^
*Lb. bulgaricus* 23	1	6.01 ± 0.04^abA^	5.89 ± 0.40^A^
	5	5.80 ± 0.14^abAB^	5.98 ± 0.04^B^
	10	5.35 ± 0.49^abB^	6.09 ± 0.12^B^

*Note:* The differences (*P* < 0.05) among samples were indicated with lower‐case letters (a, b, ab, etc.) while the differences between storage times were indicated with upper‐case letters (A, B). Values are shown as Mean ± SD.

Abbreviation: PS, Pasteurized milk.

As to TAMB, we could observe the increase in number for samples containing postbiotics of *L. plantarum* NCCB 100524 and *Lb. bulgaricus* 23. This indicates that the presence of postbiotics had a countereffect on the thriving ability of mesophilic bacteria. As shown by Ziadi et al. (2019), the increased aerobic mesophilic count in samples having postbiotics of *L. plantarum* NCCB 100524 might happen due to the neutralization of organic acids (like lactic acid) and hence, subsequent loss in their antimicrobial activity, which causes the TAMB to thrive more easily in the samples. The decrease in TAMB count during storage in samples containing postbiotics of *Lc. lactic* NCCB 100539 could be explained by the presence of various strain‐dependent bacteriocins. The results are contrary to the findings of Kaya and Şimşek (2020) for the *Lc. lactis* PFC77 strain that showed very little decrease in TAMB during fermentation. A similar result was obtained for the postbiotics of *Lb. bulgaricus* 23. Although postbiotics from *Lb. bulgaricus* 23 demonstrated an inhibiting effect on *E. coli*; the total bacterial count increased in the same sample, indicating that these postbiotics do not inhibit the growth of certain other bacteria. The increase in aerobic mesophilic bacteria is contrary to the findings of Barzegar et al. (2023), who showed a significant decrease in bacterial load by the addition of *Lactobacillus* postbiotics in various edible coatings.

## 4 Conclusion

Although probiotics have been in use for quite some time, the concept of using postbiotics is relatively new. Upon determining the negative effects of live bacteria on their use both in human consumption and in the dairy industry, in‐depth studies have begun to develop alternative applications. In the current study, postbiotics belonging to *E. faecalis* ZH‐2, *L. plantarum* NCCB 100524, *L. paraplantarum* NCCB 100523, and *Lc. lactis* NCCB 100539 was accepted as a potential candidate in terms of in vitro functional properties. As to biopreservation capacity, *E. faecium* NCCB 100527, *Lb. helveticus* 9‐B_5_, *Lb. brevis* NCCB 100526, and *Lc. lactis* NCCB 100539 was chosen as the best source for postbiotic acquisition. For these reasons, it is suggested that the preferred microbial source should be selected based on the specific desired properties of the product, such as antioxidant, antimicrobial, and/or biopreservation capacity, or higher phenolic and/or organic acid content.

## Author Contributions

**Areeba Tariq:** data curation (equal), formal analysis (equal), investigation (equal), methodology (equal), writing – original draft (equal). **Merve Nur Tahran:** formal analysis (equal), methodology (equal). **Sebnem Ozturkoglu‐Budak:** data curation (equal), conceptualization (equal), investigation (equal), supervision (equal), funding acquisition (lead), writing – review and editing (lead).

## Conflicts of Interest

The authors declare no conflicts of interest.

## Data Availability

Data will be made available on request.
